# Potential marker genes for chronic obstructive pulmonary disease revealed based on single-cell sequencing and Mendelian randomization analysis

**DOI:** 10.18632/aging.205849

**Published:** 2024-05-23

**Authors:** Gang Sun, Yun Zhou, Xiaoxiao Han, Xiangqian Che, Shuo Yu, Di Song, Feifei Ma, Lewei Huang

**Affiliations:** 1 General Hospital of Northern Theater Command, Shenyang 110000, Liaoning, China; 2 East China Normal University Wuhu Affiliated Hospital (The Second People’s Hospital of Wuhu City), Wuhu 241000, Anhui, China; 3 The Second People's Hospital of Hefei, Hefei Hospital Affiliated to Anhui Medical University, Hefei 230022, Anhui China

**Keywords:** chronic obstructive pulmonary disease, genetics, single-cell RNA sequencing, quantitative trait loci, mendelian randomization analysis

## Abstract

Background: Progress is being made in the prevention and treatment of chronic obstructive pulmonary disease (COPD), but it is still unsatisfactory. With the development of genetic technology, validated genetic information can better explain COPD.

Objective: The study utilized scRNA-seq and Mendelian randomization analysis of eQTLs to identify crucial genes and potential mechanistic pathways underlying COPD pathogenesis.

Mehods: Single-cell sequencing data were used to identify marker genes for immune cells in the COPD process. Data on eQTLs for immune cell marker genes were obtained from the eQTLGen consortium. To estimate the causal effect of marker genes on COPD, we selected an independent cohort (ukb-b-16751) derived from the UK Biobank database for two-sample Mendelian randomization analysis. Subsequently, we performed immune infiltration analysis, gene set enrichment analysis (GSEA), and co-expression network analysis on the key genes.

Results: The 154 immune cell-associated marker genes identified were mainly involved in pathways such as vacuolar cleavage, positive regulation of immune response and regulation of cell activation. Mendelian randomization analysis screened four pairs of marker genes (GZMH, COTL1, CSTA and CD14) were causally associated with COPD. These four key genes were significantly associated with immune cells. In addition, we have identified potential transcription factors associated with these key genes using the Cistrome database, thus contributing to a deeper understanding of the regulatory network of these gene expressions.

Conclusions: This eQTLs Mendelian randomization study identified four key genes (GZMH, COTL1, CSTA, and CD14) causally associated with COPD, providing new insights for prevention and treatment of COPD.

## INTRODUCTION

Chronic obstructive pulmonary disease (COPD) is a respiratory disease characterized by persistent airway inflammation and is now the third leading cause of death worldwide [1]. Chronic inflammation in the periphery of the bronchi and fine bronchioles could lead to lung tissue destruction, fibrosis and emphysema. Continued progression of COPD leads to deterioration of the patient’s condition and a corresponding increase in healthcare costs and mortality [2]. However, to date, there is no satisfactory therapeutic regimen that can slow the progression of COPD or reduce mortality [3], and one important reason for this is that the underlying pathogenesis of COPD is complex. Consequently, identification of new markers is important for the prognosis and management for COPD patients.

In patients with COPD, the exacerbation of lung tissue damage and subsequent lung remodeling due to persistent chronic inflammation is closely associated with an aberrant immune response. In this process, a variety of immune cells involved in both innate and adaptive immunity play important roles [4]. For example, macrophages and neutrophils in the alveoli could secrete a variety of proteases, cytokines, and chemokines thereby leading to destruction of the alveolar wall and thus exacerbation of emphysema [5, 6]. Adaptive immunity is a process that occurs when innate immunity is activated and involves the T-cell population responsible for early warning effects and the B-cell population that produces antibodies. Relevant studies have shown that fine bronchial damage and remodeling of alveolar tissue in lung tissue were associated with over-infiltration of CD4, CD8 and B cells [7]. In conclusion, the persistence of chronic inflammation influences the course of COPD and this inflammatory response is closely associated with an increase in a variety of immune cells [8].

With the development of bioinformatics technology, there are more possibilities to understand the mechanisms of disease progression. Traditional transcriptome sequencing measures the average expression of individual genes in a large population of cells and is primarily used to study differential expression between tissues [9]. It is therefore difficult to detect molecular differences that are only relevant to specific cell types, especially when gene expression is in low abundance in some specific cell types [10]. In recent years, technologies that can be used to assess the expression of gene profiles in individual cells (single-cell RNA sequencing) have been developed [11]. Single-cell RNA sequencing (scRNA-seq) is capable of assessing the amount of gene expression within a single cell, not only to detect rare or low abundance cell populations, but also to label previously unknown cell types or subtypes [12, 13]. In addition to this, scRNA-seq could help researchers to understand cell-to-cell information transfer, thus enabling the exploration of pathological mechanisms of diseases and the identification of new diagnostic markers or new therapeutic targets at the single-cell level [14–16]. For example, Li et al. noted in their study that scRNA-seq could be used to identify transcriptional changes and levels of individual proteins that may contribute to the development of emphysema in a cell-type specific manner [17]. Pei et al. demonstrated changes in immune cell subtypes during the progression of COPD by using peripheral blood mononuclear cells from patients with COPD for scRNA-seq [18]. However, the current types of scRNA-seq studies were mainly cross-sectional studies, which were designed to explore potential associations between genes and diseases rather than to establish causal relationships [19]. If the causal relationship between markers labeled by scRNA-seq and disease occurrence could be verified, it would not only save the cost of experiments but also provide a clearer direction for research.

In order to explore the causal associations between risk factors and diseases, Mendelian randomization (MR) analysis has attracted the attention of researchers as a new epidemiological method [20]. MR analysis is based on the principle of random assignment of genetic variants, with single nucleotide polymorphisms (SNPs) as instrumental variables (Ivs) representing the characteristics of interest, which can be used to validate causality with diseases. Thus, the combination of MR analysis with scRNA-seq results may provide greater insight into the mechanisms of development of certain diseases. For example, Wu et al. identified in their study that risk genes for schizophrenia were highly expressed in specific neuronal cells and clarified the causal relationship [21]. Indeed, expression quantitative trait loci (eQTLs) in lung tissues have gained some degree of discovery in the pathogenesis of COPD [22, 23]. However, association analyses of eQTLs based on scRNA-seq results and precision locus analysis combined with genome-wide association study (GWAS) data were still limited. Therefore, we propose in this study to utilize GWAS data on COPD with the results of scRNA-seq for a comprehensive analysis, aiming to provide new insights into the pathogenesis of COPD.

## MATERIALS AND METHODS

### Data sources

### Exposure data

The eQTLs data in this study were obtained from the eQTLGen Consortium (https://www.eqtlgen.org) database. The eQTL Gen Joint Research Program aims to analyze gene expression levels in peripheral blood and gain insight into the genetic basis of complex traits. More details about the eQTLGen Consortium data can be found in a previous report [24].

### Outcome data

The GWAS data for those diagnosed with COPD come from UK Biobank. UK Biobank contains genetic and health information on over 500,000 participants of European descent. We selected an independent cohort (ID: ukb-b-16751) containing 3871 COPD patients and 459,139 controls. The outcome data are publicly available and more details can be found at https://gwas.mrcieu.ac.uk/datasets/ukb-b-16751/.

### RNA-sequencing data

The GEO (Gene Expression Omnibus) database (https://www.ncbi.nlm.nih.gov/geo/) is a gene expression database established and maintained by the National Center for Biotechnology Information (NCBI). In this study, we obtained serial matrix data from this publicly available database containing lung tissue samples from COPD cases (GSE57148) annotated as GPL11154. Expression profiling data from a total of 189 groups of patients were included, including 91 controls and 98 COPD patients. In addition, we also obtained data from scRNA-seq of lung tissue samples from 3 COPD patients (GSE167295) for analysis.

### Gene set

The gene set related to immunization in this study was obtained from the GeneCards (https://www.genecards.org) database.

### Analysis methods for single-cell sequencing

Firstly, gene expression profiles of scRNA-seq samples were read using the Seurat package and screened for aberrantly expressed samples (nFeature_RNA > 100 and percent.mt < 10). Subsequently, the resulting information was normalized with homogenization and subjected to principal component analysis. The optimal number of principal components (PCs) was observed by ElbowPlot curves (n=12), and the positional relationship between different clusters was subsequently investigated using the t-distributed stochastic neighbor embedding (TSNE) algorithm. To add annotations to the clusters, we used the annotation file HumanPrimaryCellAtlasData, which was included in the celldex package, to label each cluster as a cell type that is closely related to the formation of the disease. Finally, the labeled genes corresponding to each cell subtype were obtained by setting the thresholds of logfc and minimum pct to 1 and 0.45, respectively, in the FindAllMarkers function. The unique marker genes associated with each cell subtype were screened using p_val_adj<0.05 and |avg_log2FC|>1.5 as conditions.

### Gene functional enrichment analysis

Through the Metascape database (https://www.metascape.org), we have functionally annotated key genes in order to further explore their interrelationships. Based on this, we would conduct Genomics Ontology (GO) and Kyoto Encyclopedia of Genes and Genomes (KEGG) pathway studies. Minimum number of overlaps (Min overlap) greater than or equal to 3 and p-value less than or equal to 0.01 were judged to be statistically significant.

### Mendelian randomization analysis

We performed a MR analysis using the MR Base database and GWAS database (https://gwas.mrcieu.ac.uk/). The Mendelian randomization analysis in this study was performed on the basis of satisfying the assumptions of association, independence and exclusivity. In the association analysis, SNPs significantly associated with each locus first needed to be screened as variable instruments (*P*<10^-8^). Subsequently in linkage disequilibrium (LD) analysis, we retained specific SNPs based on R^2^<0.001 (using a clustering window of 10,000 kb) (*P*<5×10^-8^). Subsequently, we assessed the causal effect between genotype and disease using four statistical methods (inverse variance weighting, MR Egger, weighted median, and weighted mode). Specifically, inverse variance weighted (IVW) is designed to utilize the Wald value for each SNP in a pooled analysis. MR-Egger is able to assess whether genetic variation has multiple effects on the outcome that are on average non-zero (directional multiple effects), as well as to provide causal effects under the assumption of instrumental strength independent of direct effect (InSIDE) estimation. The weighted median model is able to correctly estimate causality in up to 50% of cases where the Ivs are invalid, and the Weighted mode approach has greater ability to detect causal effects because of smaller biases and lower type I error rates. By evaluating each SNP, we would be able to further screen and validate whether there is a true causal relationship among them. We analyzed each SNP in the screened causal relationships by independent assessment using Wald ratio to screen and validate the results. Finally, we used the leave-one-out analysis to verify the independence of the screened causal relationships.

### Co-location analysis

We performed co-localization analyses using the coloc algorithm on eQTL summary data and GWAS data from COPD. The 100-kilobase region around the index SNP was used to calculate the posterior probability. In the results, H3 indicated the posterior probability that two traits were correlated but had different causal variants; H4 indicated the posterior probability that two traits were correlated and shared a single causal variant.

### Analysis of immune cell infiltration

To assess the relative proportion of 22 types of immune infiltrating cells, we employed the CIBERSORT algorithm to analyze RNA-seq data from different sub-groups of COPD patients. Subsequently, we evaluated the correlation between gene expression levels and immune cell content using Spearman correlation analysis. P-value less than 0.05 was considered statistically significant.

### Gene set enrichment analysis

To identify genes whose gene expression profiles differed between the high- and low-expression groups in COPD patients, we performed gene set enrichment analysis (GSEA; http://www.broadinstitute.org/gsea). The numbers of maximum and minimum gene sets were set as 500 and 15 as filtering conditions, respectively. The selected gene sets were ranked 100 times and the final results were obtained based on a *P*-value of <0.05 and a false discovery rate (FDR) value of 0.25 [25].

### Regulatory network of target genes

Cistrome DB is currently a more comprehensive database for studying ChIP-seq and DNase-seq, encompassing a total of 30,451 human and 26,013 mouse samples of transcription factors, histone modifications, and chromatin. In this study, we probed the regulatory relationships between transcription factors and target genes through the Cistrome DB database, with the genome file set to hg38 and the transcription start site set to 10kb, all visualized via Cytoscape.

### Statistical analysis

All data analyses were conducted in R version 4.0. MR analysis follows three fundamental assumptions: [1] the correlation assumption, stating that the instrumental variable is closely related to the exposure factor, but not to the outcome; [2] the independence assumption, meaning that the instrumental variable and the confounder have no relationship; and [3] the exclusion assumption, stating that the instrumental variable only affects the outcome through the exposure factor. If the instrumental variable affects the outcome through other pathways, it is considered to be genetic pleiotropy. A significance threshold of P<0.05 was set for statistical differences.

### Data availability statement

The original contribution to the study was included in the article/supplementary material. For further inquiries, please contact the corresponding author.

## RESULTS

### Preprocessing results of single-cell sequencing data expression profiles

This study includes the results of scRNA-seq of lung tissue from 3 patients with COPD. To proceed with the subsequent analysis, we only retained cells with nFeature_RNA greater than 100 and percent.mt less than 10. A total of 13,838 cells with expression levels of features were included for further analysis (Supplementary Figure 1A, 1B). We displayed the gene expression patterns across samples and marked the top 5 genes with the highest standardized variance (Supplementary Figure 1C).

### Single-cell sample subtype clustering analysis

We utilized Principal Component Analysis (PCA) in order to reduce the dimensionality of the differential genes. The results suggested that the 20 genes that were presented scored differently on different dimensions. (Supplementary Figure 2A). However, when we analyzed the PCA dimensionality reduction among samples, we found that the overall difference was not significant (Supplementary Figure 2B). Through observing the ElbowPlot, we found that the optimal pc number was 12 (Supplementary Figure 2C, 2D), and finally obtained 18 cell subtypes through TSNE method (Supplementary Figure 2E).

### Annotation of cluster subtypes

We used HumanPrimaryCellAtlasData as an annotation dataset and used the SingleR software package to annotate each cell subtype. The 18 clusters were annotated to B-cell, Endothelial cells, Epithelial cells, Macrophage, Monocyte, NK cell, T-cells, and Tissue stem cells (Supplementary Figure 2F, 2G). Finally, we extracted a total of 411 marker genes for cell subtypes from the single-cell expression profiles using the FindAllMarkers function (Supplementary Table 1).

### Marker gene function analysis

**Figure 1:**
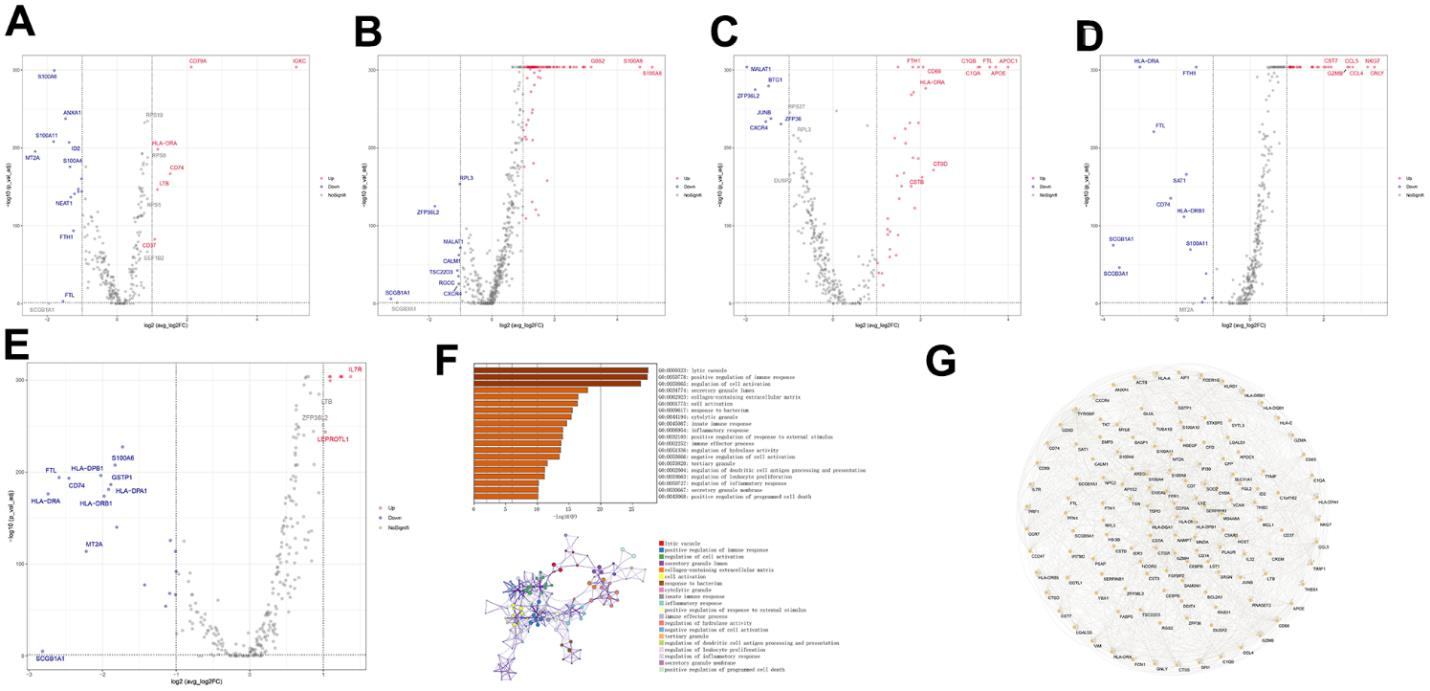
**Differential gene expression and functional analysis of immune-related cell subtypes in COPD samples.** (**A**–**E**) Volcano plots of the differential genes of the five immune cells, B_cell, Macrophage, Monocyte, NK_cell, and T_cells, respectively, in COPD samples, according to the order. (**F**) Functional enrichment analysis of the differential genes. (**G**) Differential gene protein-protein interactions network.

In the functional analysis of marker genes, we first compared genetic differences between cell subtypes associated with the immune system (B_cells, macrophages, monocytes, NK_cells, and T_cells) and identified 154 marker genes (Figure 1A–1E). To further explore the functions of these marker genes, we utilized the Metascape database to conduct a pathway analysis. The results of this analysis suggested that these marker genes were primarily enriched in pathways related to the lytic vacuole, the positive regulation of immune response, and the regulation of cell activation (Figure 1F). Additionally, we utilized Cytoscape software to visualize the protein interaction network of the gene sets (Figure 1G).

### Mendelian randomization analysis of marker genes

**Figure 2:**
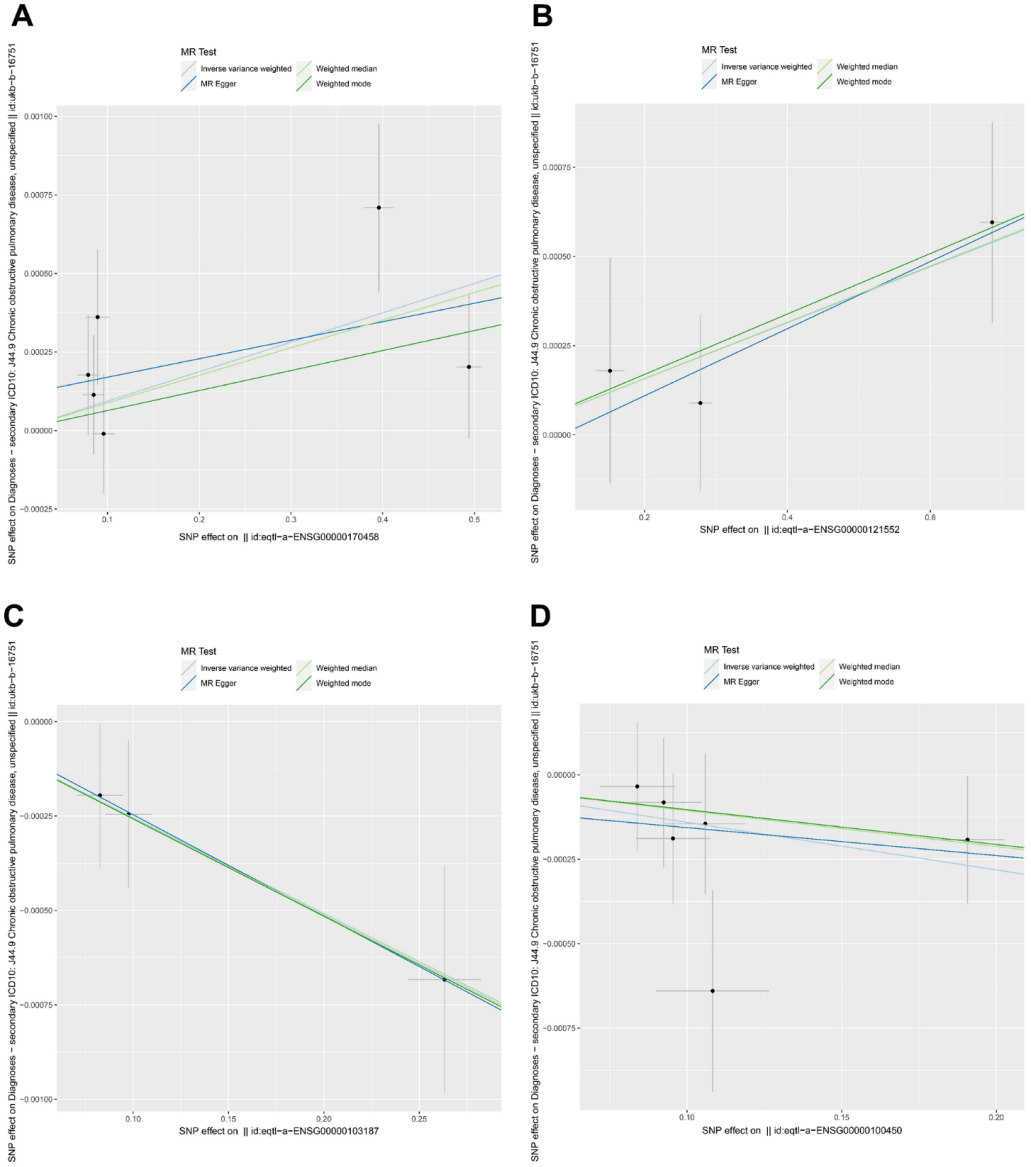
**Mendelian randomization analysis between key genes and COPD.** (**A**–**D**) Genes represented were CD14, CSTA, COTL1, and GZMH, respectively. Different colors indicated different statistical methods, and the slopes of the lines denoted the causal effect of each method, respectively.

**Figure 3:**
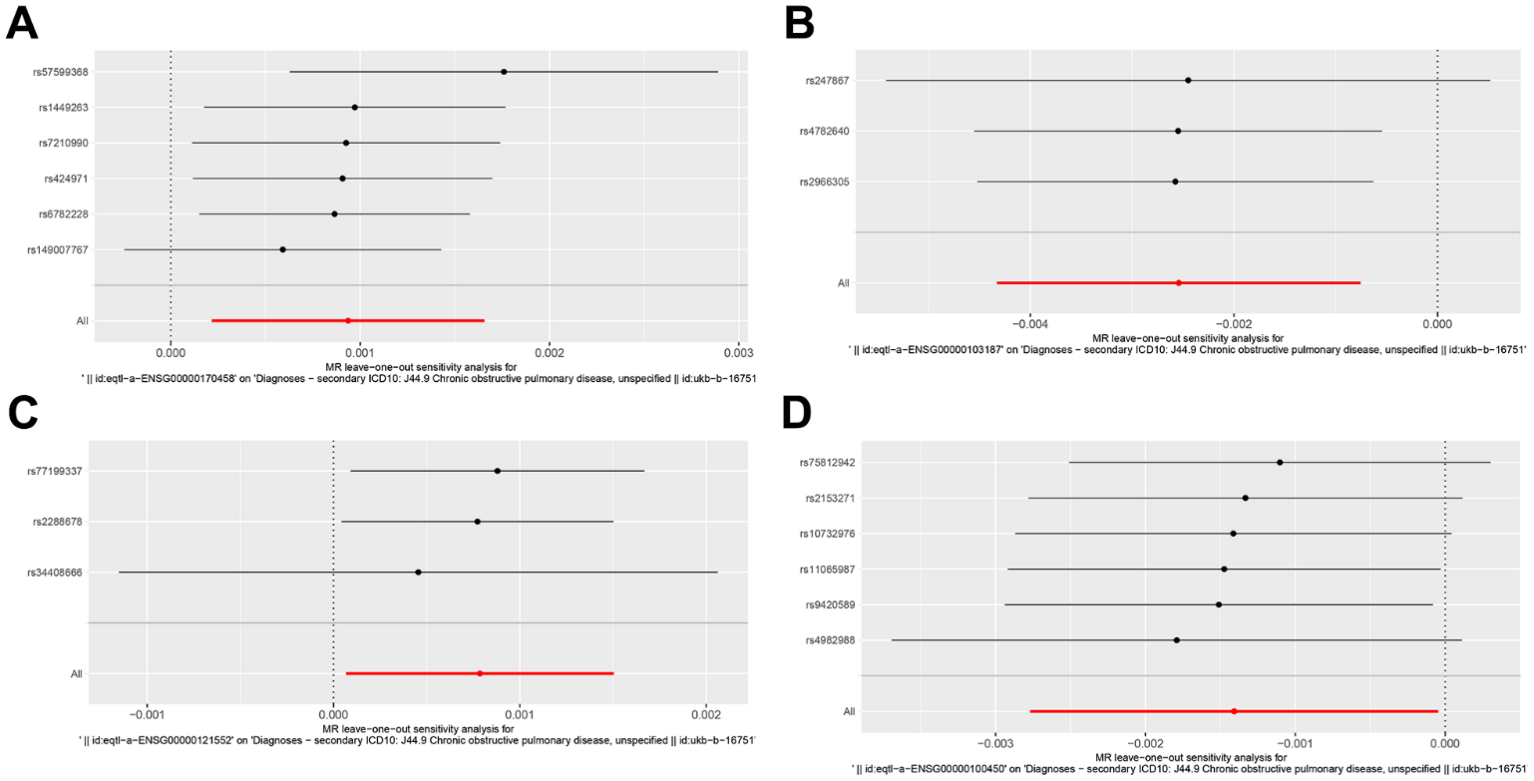
**Forest plot of key genes corresponding to SNPs tested by leave-one-out analysis.** (**A**–**D**) Respectively represented by the genes CD14, COTL1, CSTA and GZMH.

**Figure 4:**
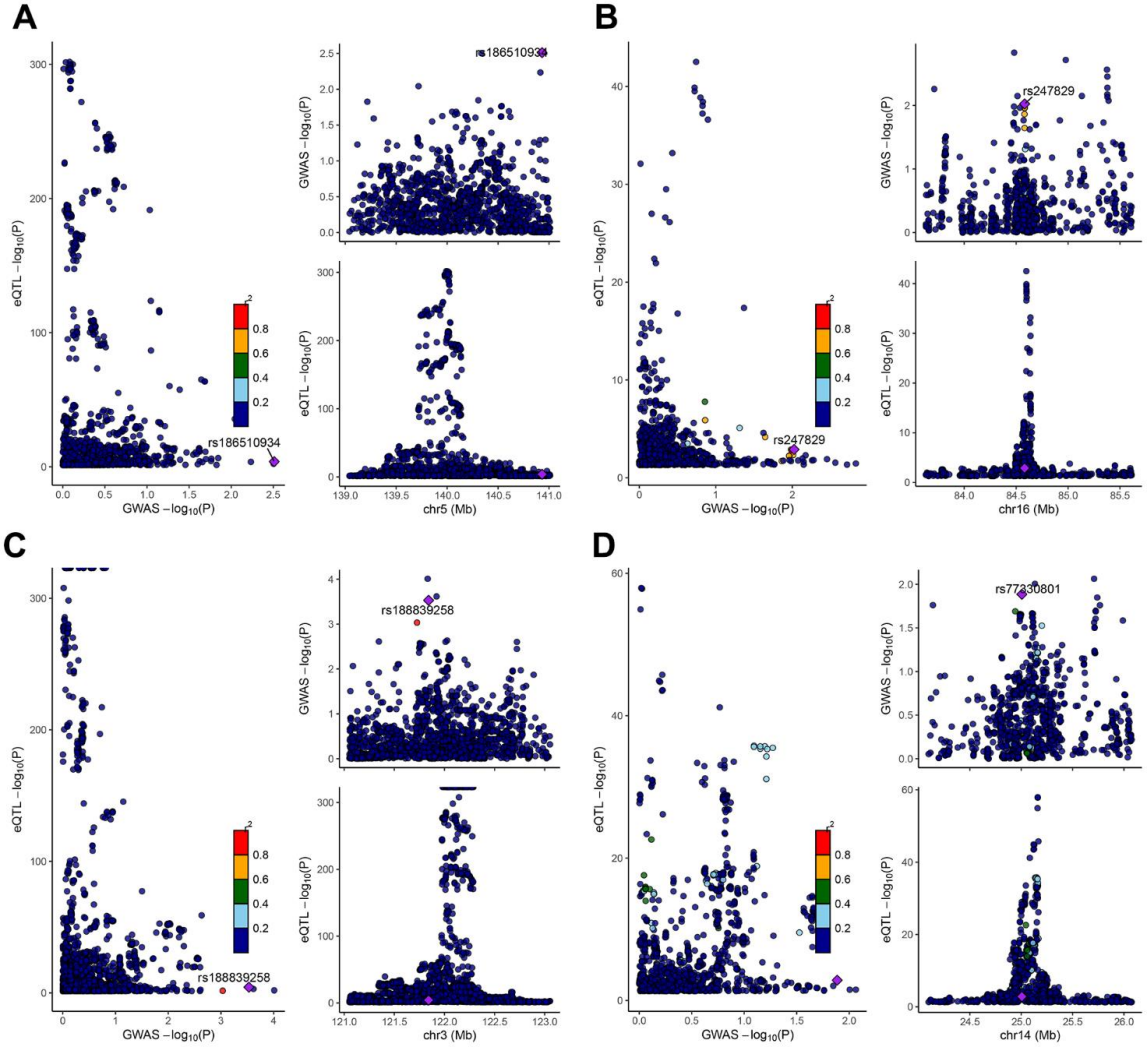
**Co-localization analysis between SNPs of key genes and GWAS data from COPD patients.** The genes represented from (**A**–**D**) were CD14, COTL1, CSTA, and GZMH, respectively. The X-axis indicated the P-value of GWAS, and the Y-axis indicated the P-value of eqtl.

We performed a Mendelian randomization analysis of marker genes to further identify key genes marked by marker genes that would have an impact on COPD. Using the COPD meta-analysis summary statistics (id: ukb-b-16751 with 459,139 controls and 3,871 cases), we extracted 120 pairs of marker genes causally associated with COPD (Supplementary Table 2). Subsequently, by MR analysis, we screened 4 pairs of marker genes that were causally associated with positive eQTL results (Figure 2A–2D, p-value of IVW less than 0.05). These 4 pairs of marker genes were GZMH, COTL1, CSTA, and CD14. The effect values corresponding to these 4 pairs of marker genes were COTL1 (OR= 0.997; 95% CI: 0.996 - 0.999; *P* = 0.005), GZMH (OR= 0.999; 95% CI: 0. 997-0.99995; *P* = 0.043), CSTA (OR= 1.001; 95% CI: 1.000-1.002; *P* = 0.032), and CD14 (OR= 1.001; 95% CI: 1.000-1.002; *P* = 0.011), as described in more details in Supplementary Table 3. These results suggest that CSTA and CD14 could be high-risk factors for COPD; while COTL1 and GZMH might be low-risk factors for COPD. In addition, we performed sensitivity analysis to determine the reliability of the causality for the four genes. The results showed that the exclusion of any of the SNPs had a small effect on the overall error, suggesting that the selected 4 pairs of key genes have a robust causal association with COPD (Figure 3). Finally, we performed co-localization analysis of these four genes at the eQI-GWAS level (Figure 4). We found that COTL1 was associated with COPD and shared genetic loci with single mutations (Figure 4B and Supplementary Table 4).

### Clinical predictive value for key genes

**Figure 5:**
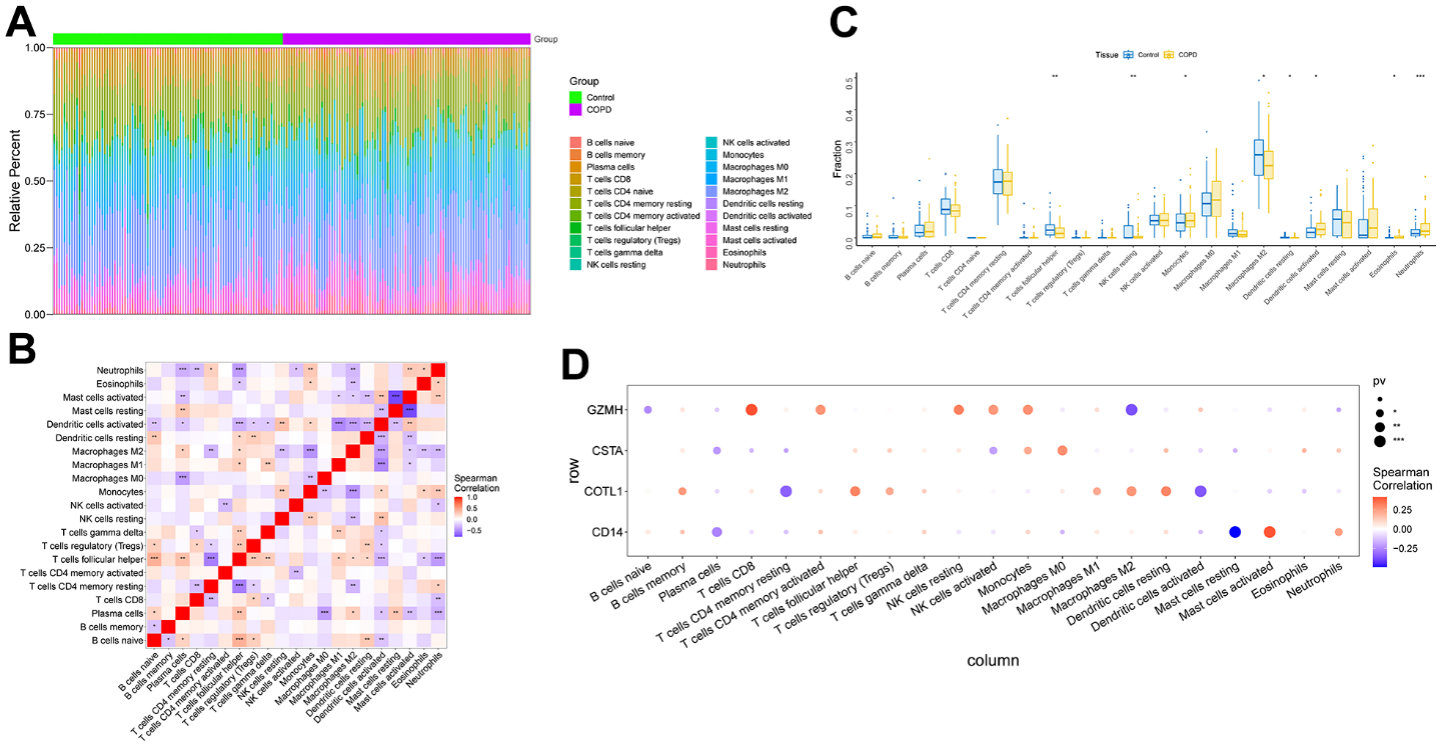
**Analysis of immune cell infiltration in COPD samples.** (**A**) Relative percentages of immune cell subpopulations. (**B**) Correlation between immune cell subpopulations, where red represents positive correlation and blue represents negative correlation. (**C**) Differences in immune cell content between COPD and control samples. (**D**) Correlation between 4 key genes and immune cells. Where red represents a positive correlation and blue represents a negative correlation, the larger black solid circle represents a more statistically significant difference.

**Figure 6:**
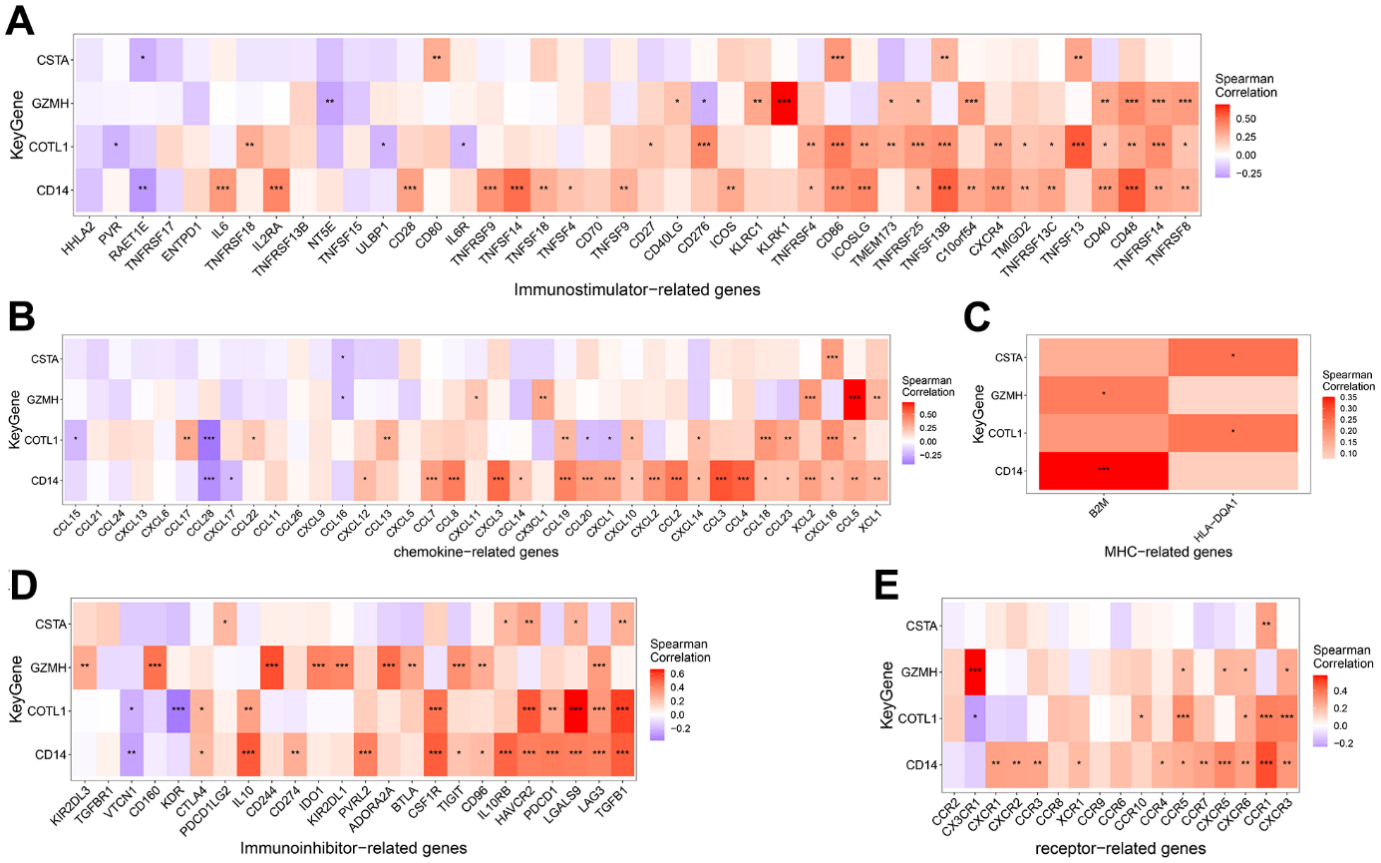
Correlation between key genes and Immunoinhibitor (**A**), Chemokine (**B**), MHC (**C**), Immunostimulator (**D**) and Receptor related immunogenes (**E**).

The microenvironment is mainly composed of immune cells, extracellular matrix, various growth factors, inflammatory factors, and special physicochemical features, etc. The microenvironment significantly influences the diagnosis of the disease, survival outcome, and sensitivity of clinical treatment. By analyzing the relationship between gene expression levels and immune infiltration, the potential molecular mechanisms by which the expression levels of key genes influence the development of COPD can be further explored. Our results showed that the distribution of immune levels of different immune factors in the samples was not entirely consistent (Figure 5A). There were several significant correlations among the immune factors (Figure 5B). monocytes, Dendritic cells activated, etc. were significantly higher in COPD samples than in control samples, while T cells follicular helper, Macrophages M2, etc. were significantly lower than in control samples (Figure 5C). The gene GZMH was significantly positively correlated with T cells CD8, NK cells resting, etc., and significantly negatively correlated with B cells naive, Macrophages M2; the gene COTL1 was significantly positively correlated with Macrophages M2, Dendritic cells resting, etc., and significantly positively correlated with T cells CD4 memory resting, Dendritic cells activated, etc.; gene CSTA is significantly positively correlated with Monocytes, Macrophages M0, etc., and negatively correlated with Plasma cells, NK cells activated, etc.; gene CD14 is significantly correlated with Mast cells activated, Neutrophils significantly positively correlated, and significantly negatively correlated with Plasma cells, Mast cells resting (Figure 5D). We obtained the correlations between these key genes and different immune factors, including immunomodulators, chemokines and cellular receptors from the TISIDB database, and the results showed that the key genes were significantly correlated with several immune factors (Figure 6).

### Disease gene expression levels

**Figure 7:**
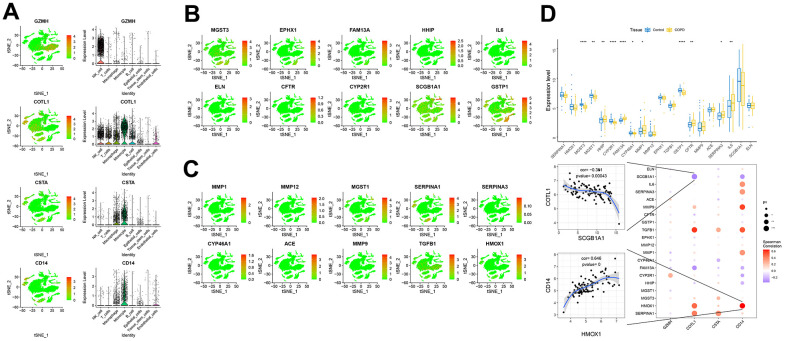
**Expression of key genes in single cells.** (**A**–**C**) Expression of key genes and disease-causing genes in cells. (**D**) The upper panel represented differences in the expression of disease-regulated genes, with control patients in blue and disease patients in yellow. The lower panel represented the pearson correlation analysis between key genes and disease genes. Blue color represents negative correlation and red color represents positive correlation.

We obtained the disease genes associated with COPD through the GeneCards database (https://www.genecards.org/), and we analyzed the expression levels of four key genes (Figure 7A–7C) and the expression levels of the top 20 genes with the highest relevance score. We found that the expression levels of key genes were significantly correlated with the expression levels of several disease-related genes, including CD14, which was significantly positively correlated with HMOX1 (correlation coefficient=0.646), and COTL1, which was significantly negatively correlated with SCGB1A1 (cor=-0.351) (Figure 7D). Subsequently, we analyzed the expression of these four key genes and the top 10 immune genes with the highest relevance score at the single-cell level, and we found that the key genes were co-expressed with several immune genes at the single-cell level (Supplementary Figures 3–6).

### Potential signaling mechanisms and regulatory networks for key genes

**Figure 8:**
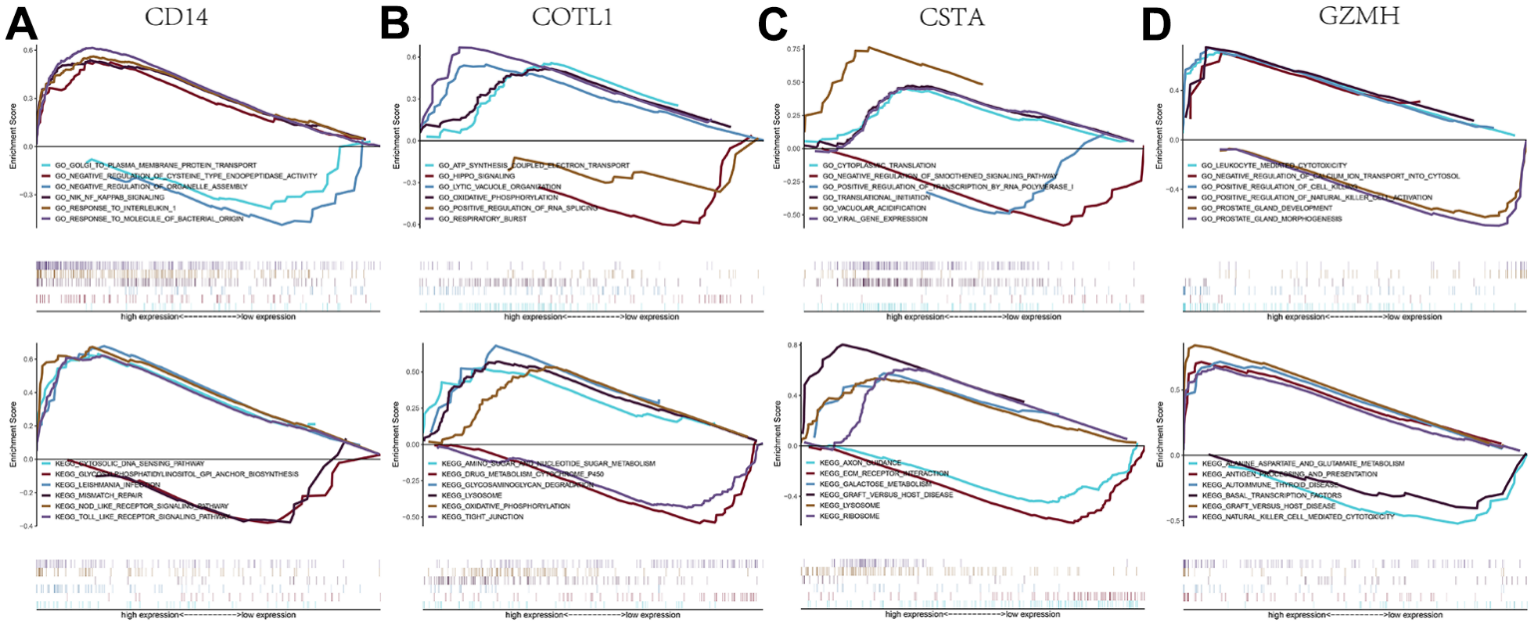
**GSEA analysis of key genes.** (**A**–**D**) GO and KEGG signaling pathways involved in different key genes.

We performed an in-depth enrichment analysis of four key genes to explore the relevant signaling pathways by which they may influence disease progression. For example, we found that the main pathways for gene ontology (GO) enrichment of GZMH gene were “LEUKOCYTE MEDIATED CYTOTOXICITY, NEGATIVE REGULATION OF CALCIUM ION TRANSPORT INTO CYTOSOL, etc” and the pathways for KEGG enrichment mainly included “ALANINE ASPARTATE AND GLUTAMATE METABOLISM, ANTIGEN PROCESSING AND PRESENTATION, etc”. The enrichment results for all four key genes were shown in Figure 8. Subsequently, we compiled these four key genes into a gene set for this analysis to further investigate the transcriptional regulatory networks encompassing these genes. Using the Cistrome DB online database, we identified the transcription factors associated with the key genes.

**Figure 9:**
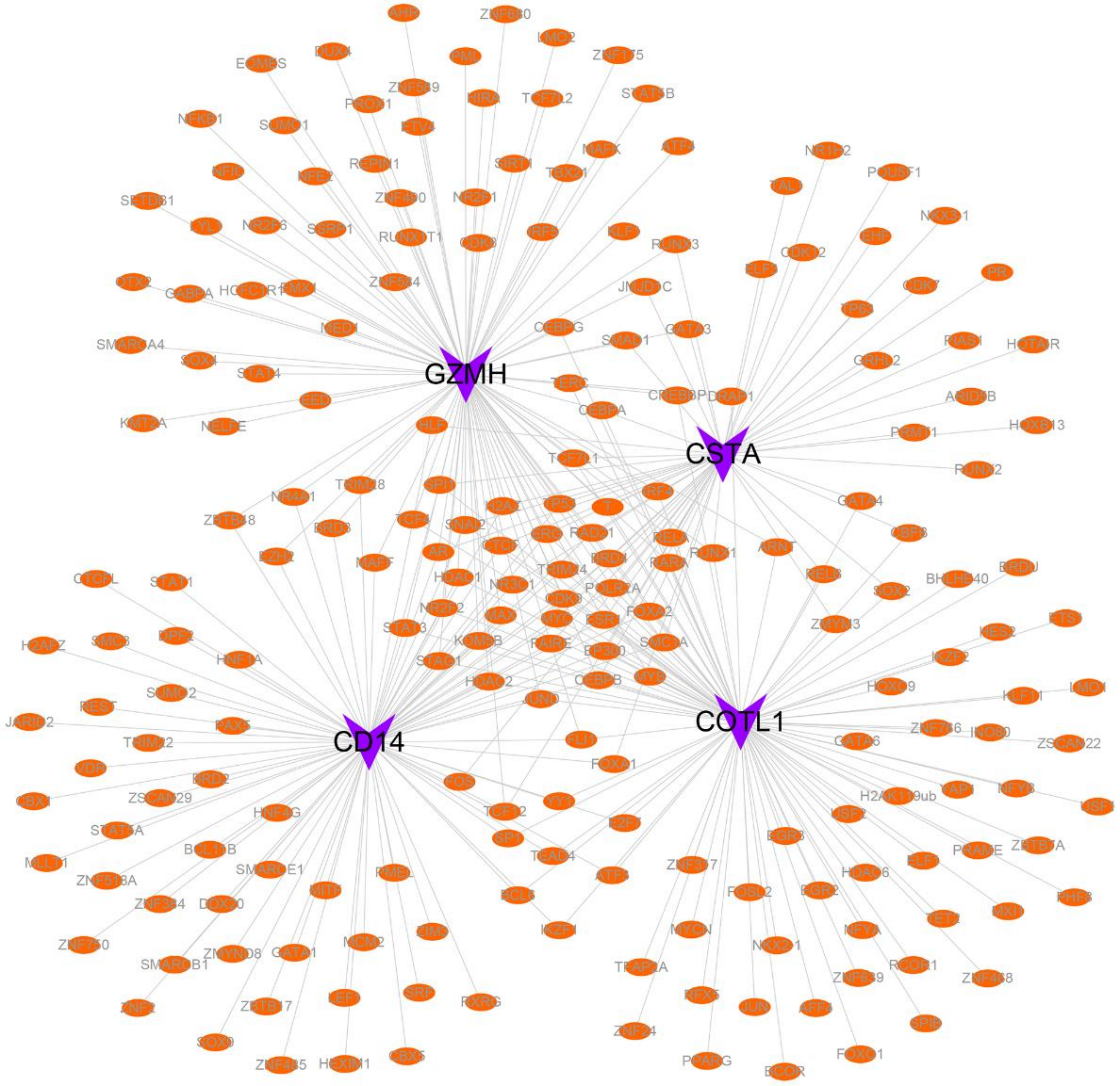
**Transcriptional regulatory networks of key genes.** Purple indicates mRNAs and orange indicates transcription factors.

Specifically, 86 transcription factors were predicted to be associated with CD14, 93 with COTL1, 54 with CSTA, and 94 with GZMH. Finally, we employed Cytoscape software to visualize the transcriptional regulatory networks of COPD-related key genes (Figure 9).

## DISCUSSION

Although smoking is widely recognized as a key factor in the development of COPD, a puzzling phenomenon is that the disease may continue to progress even when COPD patients successfully quit smoking. This phenomenon has prompted researchers to delve deeper into the genetic factors of COPD. Currently, more and more researchers tend to regard COPD as a disease with a genetic basis. Recent breakthroughs in genomic research worldwide have led to the discovery of numerous genes associated with the development of COPD, providing new clues to the pathogenesis of the disease [26–28]. Various GWAS studies have also suggested that the pathogenesis of COPD is not caused by a single genetic variant, but rather by a disruption in the balance of the biological network consisting of genes and proteins, leading to intricate changes in the pathological processes of the disease. The exploration of the pathogenesis of COPD through genetic technology holds immense clinical significance. However, with the development of gene sequencing technology, it has been recognized that the regulation of numerous genes is specific to certain cells. Previous sequencing results based on a wide range of tissues may have resulted in the omission of information regarding expression quantitative trait loci (eQTLs) specific to certain cell subtypes [21]. Therefore, the utilization of scRNA-seq technology has become invaluable in the search for crucial COPD genes.

In our results, we identified 8 cell subtypes, 5 of which were immunologically related. The immunological profile of COPD during its progression remains unclear, so further studies of immune cell infiltration in COPD are still needed. The immune inflammation associated with COPD is at the heart of the disease, which is one of the reasons why current treatments have failed to stop the progression of the disease and the continued damage to lung tissue [29]. Previous studies have shown that the effector molecule functions of neutrophils and macrophages involved in chronic inflammation in COPD are suppressed [30, 31]. In addition, it has been shown that neutrophils and lymphocytes can have a synergistic effect to enhance migration toward chemokine receptor 3 (CXCR3) and chemokine ligand 5 (CCL5), leading to immune infiltration of macrophages and T cells in the lungs of COPD patients [32]. Our study showed that key genes of immune-related cell subtypes were mainly enriched in the positive regulation of inflammatory response and cell activation, which is consistent with the above findings [33].

Most importantly, our study identified four key immune-related genes based on COPD patients. GZMH, also known as granzyme H, is a member of the serine protease family [34]. Granzyme H is expressed in immune cells (mainly cytotoxic T cells and natural killer cells) and promotes the release of inflammatory cytokines and cytolytic proteins from effective immune cells thereby inducing apoptosis in target cells [34, 35]. Since an important feature of COPD is the excessive deposition and remodeling of the extracellular matrix (ECM) around small airways and can provide mechanical pathways for the infiltration and migration of harmful immune cells [29]. Previous studies have shown that expression of granzyme’s B can contribute to the degradation and remodeling of the ECM in the extracellular environment, thereby promoting the development of an emphysema phenotype [36]. A positive correlation between GZMH and inflammation was indirectly demonstrated in a clinical randomized controlled trial suggesting that the downregulation of the GZMH gene was more pronounced in COPD patients treated with oral prednisone than in other family members [37]. In summary, a limited number of studies have been reported explaining that high expression of the granzyme family in COPD is highly correlated with the severity of the disease. However, it has also been shown that GZMH levels are reduced in patients with moderate and/or severe severity when combined with acute neocoronary pneumonia (COVID) in patients with co-morbidities, even at high levels of inflammation [38]. Our findings suggested that GZMH expression may be associated with a lower risk of developing COPD. The new puzzling question is whether GZMH expression is harmful or beneficial? Or does it play different roles at different stages of COPD? Therefore, more subsequent studies are necessary.

In our study, we found that another gene associated with a low risk of developing COPD is COTL1. COTL1 is one of the proteins that encodes for the regulation of the actin cytoskeleton, which acts mainly through the innate immune pathway [39]. In our study, COTL1 was strongly positively associated with dendritic cells as well as macrophages, which is similar to previous findings. Studies on COTL1 and immune cell infiltration in COPD samples are still unknown. COTL1 has been reported mainly in tumor samples. In a study on breast cancer, investigators demonstrated that COTL1 was highly expressed in tumor tissues and positively correlated with dendritic cells and M2 macrophages [40]. The influence of innate immunity in the COPD process is complex, with multiple types of macrophages being closely associated with pro-inflammatory responses [41], but M2 macrophages are also associated with inflammatory abatement, tissue repair, and reduction of pro-inflammatory cytokines [42]. Also not to be overlooked is the role of dendritic cells as the first barrier in the face of pathogenic microorganisms [43].

Our study demonstrated 2 key genes associated with a high risk of developing COPD, including CSTA and CD14. CSTA, also known as cystatin A, has cysteine protease inhibitory effects [44]. Butler demonstrated in their study that patients with smoking and COPD have increased levels of CSTA expression and a dose-responsive relationship with the severity of the disease [45]. Although there is still no relevant study explaining whether the high expression of CSTA gene in COPD patients is associated with the infiltration of immune cells, earlier researchers have demonstrated that the local proliferation of cystatin A-producing epidermal cells is associated with a high degree of infiltration of monocytes and granulocytes in inflammatory diseases [46]. CD14 is a co-receptor of the Toll-like Receptor (TLR)-4 in the innate immune response and plays a crucial role in its ability to recognize lipopolysaccharide, pathogens and damage-associated molecular patterns on bacterial surfaces thereby promoting an immune-inflammatory response [47]. In a study by Dewhurst, lung tissues from COPD patients were labeled for macrophage subpopulations. The results showed more infiltration of abnormally sized macrophages in the lung tissue of COPD patients compared to normal lung tissue and confirmed the high expression of the CD14 gene [48].

Finally, there are several limitations of our study. First, based on two datasets obtained from the GEO database, we obtained gene expression profiles of COPD patients. However, all participants tested were male, and the COPD group had a greater age and number of years of smoking [49]. Although Mendelian randomization analysis can be effective in reducing this potential bias, our findings need to be treated with caution in nonsmoking or female COPD patients. Second, although the dataset we utilized for single-cell sequencing was able to satisfy the cell sample size used for the analysis [50], there are still limitations in the population sample size. This leads to the possibility of a mismatch in general characteristics in COPD compared to controls [17]. In conclusion, although we identified associations between four key genes and disease genes using Mendelian randomization analysis and explored potential signaling mechanisms, whether these disease genes or signaling mechanisms exist independently or ultimately have an impact on the development of COPD through complex combinations still requires further confirmation.

## CONCLUSIONS

In this study, we identified marker genes and major pathways that could be enriched for immune cell subtypes associated with COPD by analyzing scRNA-seq data. Based on Mendelian randomization analysis of eQTLs and GWAS data, the screened genes GZMH, COTL1, CSTA, and CD14 provided evidence for a causal effect on the development of COPD. The association of these four key genes with COPD-causing genes and the potential informational pathways enriched by these genes could provide additional information on the development of COPD.

## Supplementary Material

Supplementary Figures

Supplementary Table 1

Supplementary Table 2

Supplementary Table 3

Supplementary Table 4
