# Supplementary material for: Potential marker genes for chronic obstructive pulmonary disease revealed based on single-cell sequencing and Mendelian randomization analysis
Source: Aging (Albany NY). 2024 May 23;16(10):8922–43. doi: 10.18632/aging.205849 (PMC11164476; doi:10.18632/aging.205849)
Supplement: Supplementary Figures [file aging-16-205849-s001.pdf]

SUPPLEMENTARY FIGURES

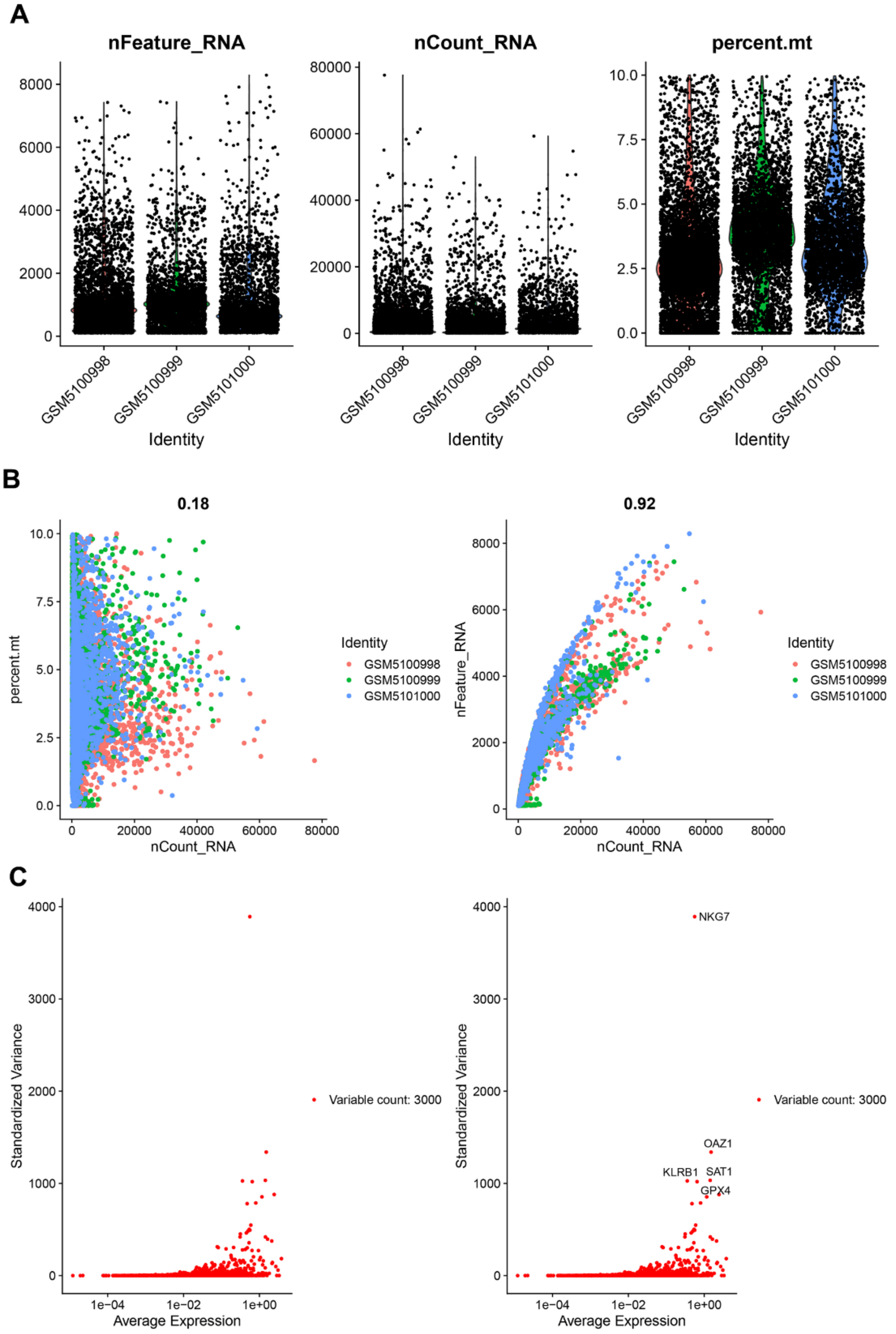

Supplementary Figure 1. Analysis of single-cell sequencing results from COPD patients. (**A**) Quality inspection of single-cell sequencing. Plots from left to right showed the number of cells, the number of genes and the sequencing depth for each sample, respectively. (**B**) Plots from left to right showed the relationship between single-cell sequencing depth and mitochondrial content (correlation coefficient of 0.18) and the number of genes (correlation coefficient of 0.92), respectively. (**C**) Genes that differed between cells, and the 5 genes with the highest standardized variance were flagged.

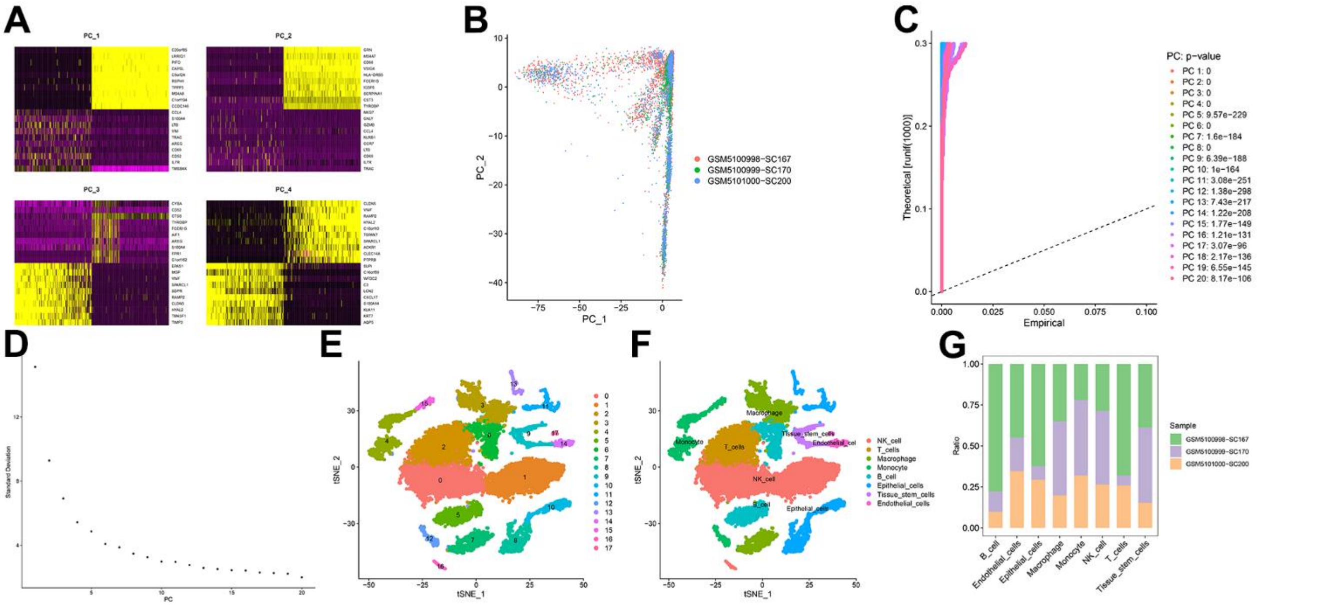

Supplementary Figure 2. Annotation of cellular subtypes of single-cell samples. (**A**) Heatmap of principal component analysis for genes from single-cell sequencing in COPD samples. (**B**) Distribution of PCs in COPD single-cell sequencing samples, where dots represent cells and different colors represent different samples. (**C**) Histogram of PCA results of COPD single-cell sequencing samples to show the P-value for each PC. (**D**) Variogram of each PC in COPD single-cell sequencing samples. (**E**) Cluster analysis of COPD single-cell sequencing samples. (**F**) Annotation of cell subtypes in COPD single-cell sequencing samples. (**G**) Difference in the percentage of cells in COPD samples that were annotated.

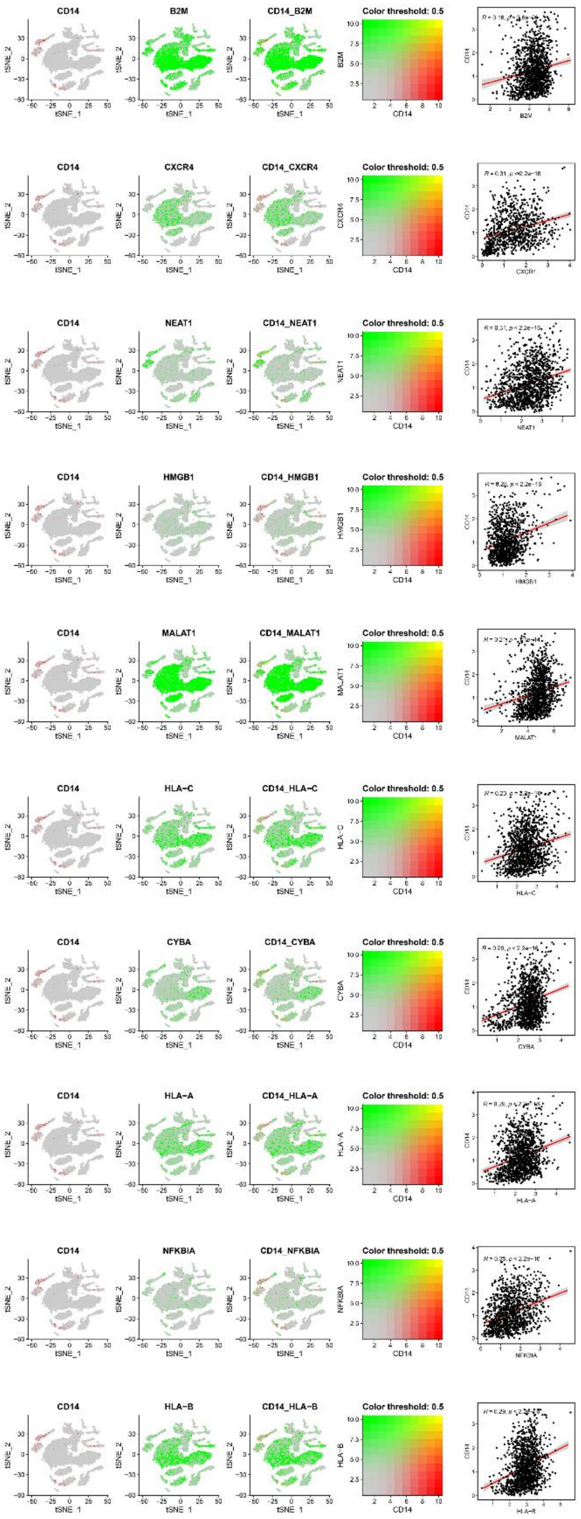

Supplementary Figure 3. Co-expression analysis of CD14 with immune genes in single-cell sequencing samples. Scatter plots represent pearson correlation analysis between co-expressed genes.

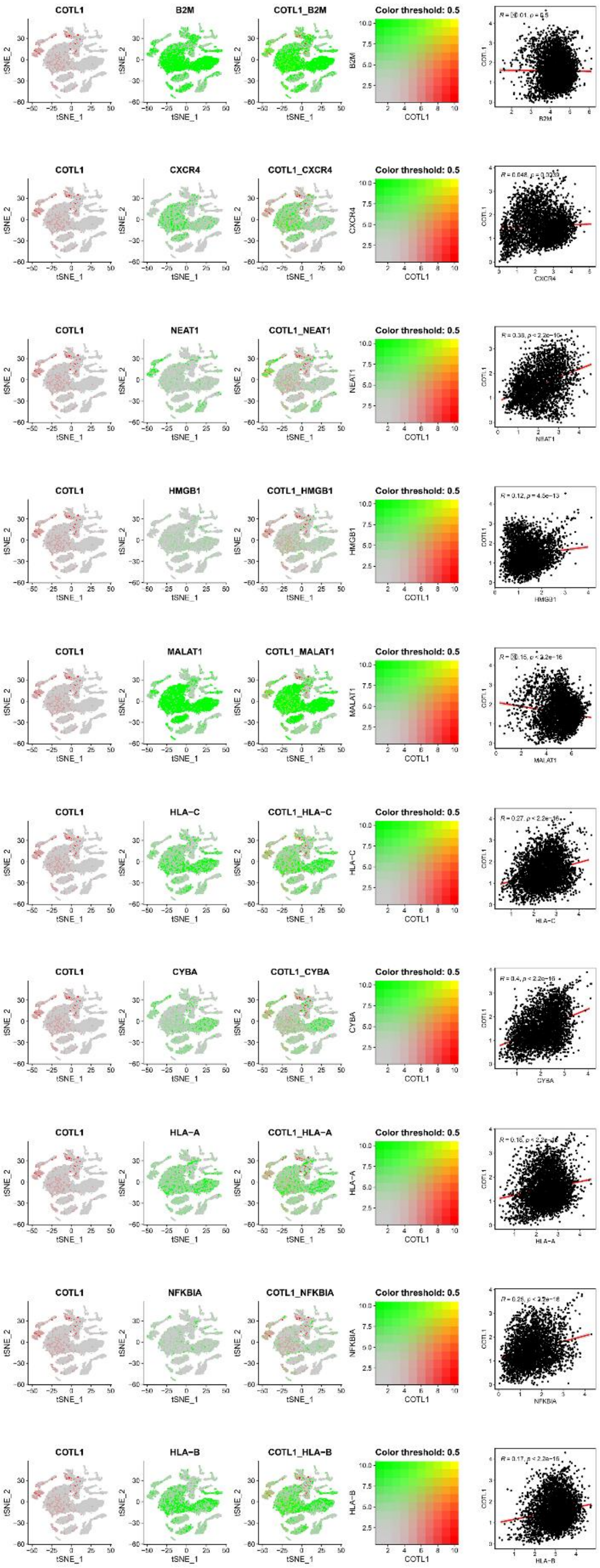

Supplementary Figure 4. Co-expression analysis of COTL1 with immune genes in single-cell sequencing samples. Scatter plots indicate the pearson correlation analysis between co-expressed genes.

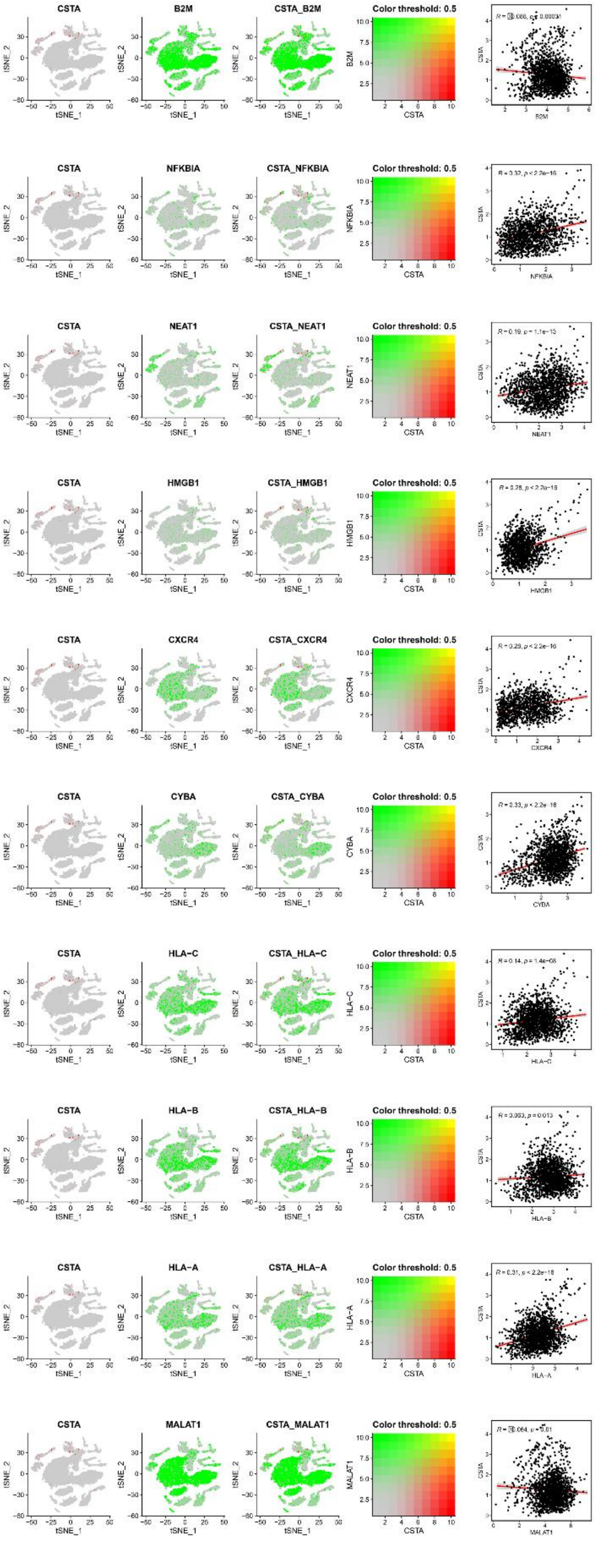

Supplementary Figure 5. Co-expression analysis of CSTA with immune genes in single-cell sequencing samples. Scatter plots indicate the pearson correlation analysis between co-expressed genes.

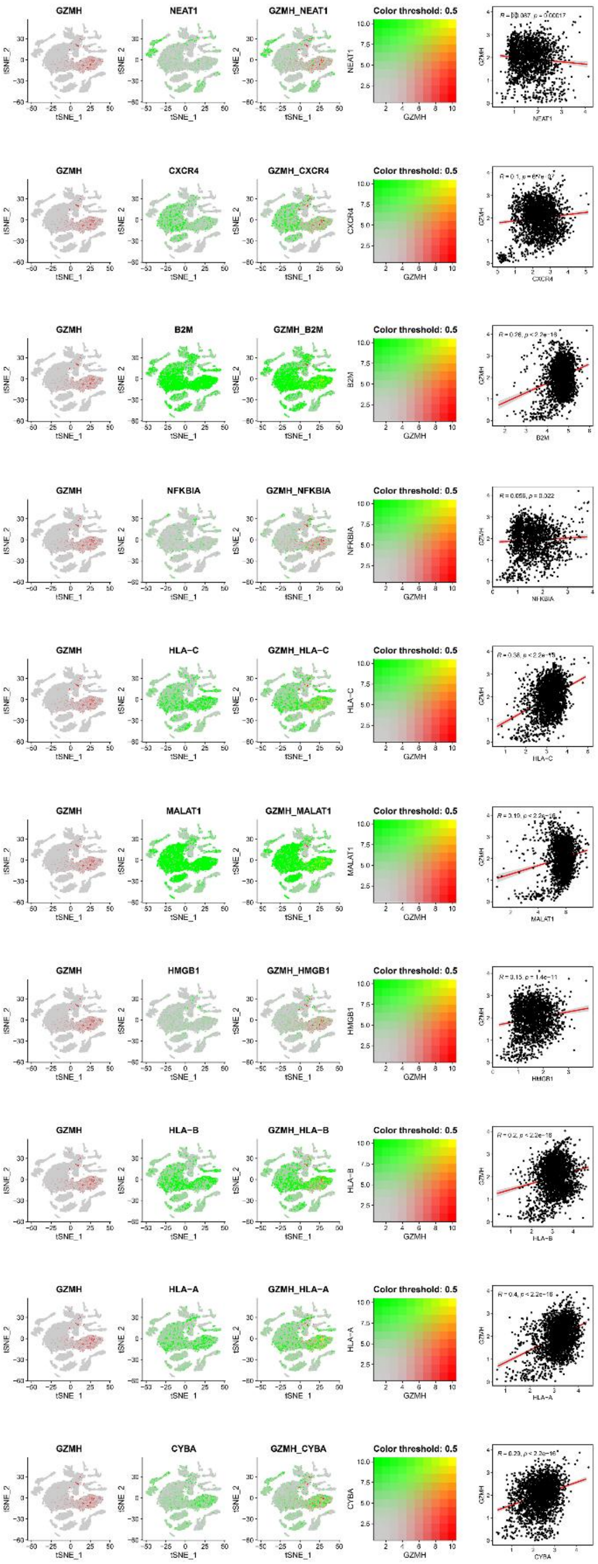

Supplementary Figure 6. Co-expression analysis of GZMH with immune genes in single-cell sequencing samples. **Scatter plots indicate the pearson correlation analysis between co-expressed genes.**
